# An analysis of published study designs in PubMed prisoner health abstracts from 1963 to 2023: a text mining study

**DOI:** 10.1186/s12874-024-02186-6

**Published:** 2024-03-17

**Authors:** George Karystianis, Wilson Lukmanjaya, Iain Buchan, Paul Simpson, Natasha Ginnivan, Goran Nenadic, Tony Butler

**Affiliations:** 1 School of Population Health, University of New South Wales, Sydney, Australia; 2 Institute of Population Health, University of Liverpool, Liverpool, UK; 3 School of Computer Science, University of Manchester, Manchester, UK

**Keywords:** Prisoner health research, Epidemiological criminology, Text mining, Observational research, Randomised controlled trials, Systematic reviews, Meta analysis

## Abstract

**Background:**

The challenging nature of studies with incarcerated populations and other offender groups can impede the conduct of research, particularly that involving complex study designs such as randomised control trials and clinical interventions. Providing an overview of study designs employed in this area can offer insights into this issue and how research quality may impact on health and justice outcomes.

**Methods:**

We used a rule-based approach to extract study designs from a sample of 34,481 PubMed abstracts related to epidemiological criminology published between 1963 and 2023. The results were compared against an accepted hierarchy of scientific evidence.

**Results:**

We evaluated our method in a random sample of 100 PubMed abstracts. An F1-Score of 92.2% was returned. Of 34,481 study abstracts, almost 40.0% (13,671) had an extracted study design. The most common study design was observational (37.3%; 5101) while experimental research in the form of trials (randomised, non-randomised) was present in 16.9% (2319). Mapped against the current hierarchy of scientific evidence, 13.7% (1874) of extracted study designs could not be categorised. Among the remaining studies, most were observational (17.2%; 2343) followed by systematic reviews (10.5%; 1432) with randomised controlled trials accounting for 8.7% (1196) of studies and meta-analysis for 1.4% (190) of studies.

**Conclusions:**

It is possible to extract epidemiological study designs from a large-scale PubMed sample computationally. However, the number of trials, systematic reviews, and meta-analysis is relatively small – just 1 in 5 articles. Despite an increase over time in the total number of articles, study design details in the abstracts were missing. Epidemiological criminology still lacks the experimental evidence needed to address the health needs of the marginalized and isolated population that is prisoners and offenders.

**Supplementary Information:**

The online version contains supplementary material available at 10.1186/s12874-024-02186-6.

## Background

Research conducted at the nexus between health sciences and criminology has emerged as a distinctive field often referred to as justice health research or epidemiological criminology [1]. This field seeks to apply the scientific principles and methods of health sciences to criminal justice settings by framing crime and offending as a public health issue involving the interplay between health, well-being and social and behavioural factors to explain and ultimately prevent offending and improve outcomes [2, 3]. However, the highly sensitive nature of those in the criminal justice system, particularly those detained in prisons and juvenile centres, makes population access difficult which thus, impacts on the ability to conduct high quality research in this setting. Issues such as competing time demands for and prioritization of prisoner programs and court and family visits impede prisoner access to research participation [4]. Limited funding for research, complex and multi-layered ethics approval processes, security barriers, understaffing, and staff and prisoner research “burnout”, combine to make epidemiological criminology research challenging [4]. It has been suggested that this, in turn, compromises the quality of research undertaken in the justice setting, particularly prisons, undermining the evidence base as more laborious study designs are abandoned in favour of more simplistic research [5].

Study design is defined as a specific plan or protocol that has been followed in the conduct of the study [6]. It can be classified into experimental (e.g., trials), observational (e.g., cross sectional) or secondary (e.g., systematic reviews, meta-analyses) [6]. Each of these three types follows (in theory) a set of reporting guidelines such as the STrengthening the Reporting of OBservational studies in Epidemiology (STROBE) guidelines [7], the Consolidated Standards of Reporting Trials (CONSORT) [8], the Standard Protocol Items: Recommendations for Interventional Trials (SPIRIT) guidelines in the abstract forms [9] and the Preferred Reporting Items for Systematic Reviews and Meta-Analyses (PRISMA) [10]. However, it has been suggested that the quality of studies in the justice health area remain suboptimal with calls to improve the evidence base [5, 11]. Whether this is true or not is unknown.

As more scientific literature becomes available, the task of reading, extracting and synthesising knowledge from large numbers of epidemiological studies becomes more time-consuming [12–16]. Methods which enable the automatic extraction of salient features of published research (e.g., study design) can provide a quick means of reporting on large numbers of documents by reducing the time required to detect, summarise and incorporate key information from relevant literature [18, 19].

While reviews undertaken by students and researchers prior to the conduct of research are the norm, few studies have attempted to analyse a whole discipline to investigate the quality of the peer reviewed outputs and trends over time. Several research efforts have been made to identify key information (e.g., study design, participant type, arm of intervention, confounding factors) from experimental and observational studies with varying degrees of success from health research, particularly from randomised controlled trials, which represent the gold standard for causal evidence on intervention effects [12, 14–24].

Since epidemiology is a field in which studies follow a semi-structured reporting style, with its own dictionary [6], we hypothesized that a simple text mining approach (i.e., rules that can identify targeted characteristics of interest) could provide an effective means to extract key information from text across the entire discipline. Epidemiological criminology studies and trials are indexed in bibliographical databases related to medicine which publish the abstracts of such studies. The abstracts are written in a relatively structured format within the journal’s own reporting style that aims to standardise and improve communication, making them ideal for the application of a rule-based text mining method [16, 17]. They are also publicly available in digital form and not behind a pay wall making it easy to conduct large scale research. The largest such database is PubMed, developed by the National Library of Medicine, which is part of the National Institutes of Health (NIS) and designed to provide access to millions of citations from biomedical journals [25]. PubMed has more than 34,000 published articles in the epidemiological criminology area.

In this study, we applied a rule-based method on 34,481 PubMed epidemiological criminology abstracts to investigate whether they reported the implemented research designs. The study design results were normalized to allow statistical analysis and compared against an accepted hierarchy of scientific evidence [26, 27].

## Methods

### Data

We conducted a literature search in PubMed using an expanded version of an existing query [28, 29] containing search terms related to offenders and prisons which were combined with either the Medical Subject Heading (MeSH) term “epidemiology” to capture all types of epidemiological studies or with all the available (in PubMed) publication types (e.g., meta-analysis, clinical trial) to ensure the results will return clinical trials and secondary research in this area. We also added terms related to randomization/natural experiments and synthetic control. These choices prevented articles that made only passing reference to prisoner and offender studies from entering the dataset resulting in a high-quality corpus for analysis. The search was restricted to English language articles that have an abstract and involved only human participants.

The full query was run on the 20th of July 2023:

*prison OR borstal OR jail OR jails OR gaol OR gaols OR penitentiary OR custody OR custodial OR (corrective AND (service or services)) OR ((correctional or detention) AND (centre or centres OR center OR centers OR complex OR complexes or facility or facilities)) OR (closed AND (setting)) OR prisoner OR prisoners OR incarcerated OR criminals OR criminal OR felon OR felons OR remandee OR remandees OR delinquent OR delinquents OR detainee OR detainees OR convict OR convicts OR cellmate OR cellmates OR offenders OR offender OR ((young OR adolescent) AND (offender OR offenders)) OR ((delinquent OR incarcerated) AND youth) OR (juvenile AND (delinquents OR delinquent OR delinquency OR detainee OR detainees OR offender OR offenders)) OR ((young) AND (people) AND (in) AND (custody)) OR ((justice) AND (involved) AND (youth)) OR ((incarcerated) AND (young) AND (people OR person OR persons)) OR ((juvenile OR juveniles) AND (in) AND (custody)) AND english[lang] AND (“epidemiology“[Subheading] OR “epidemiology“[MeSH Terms] OR epidemiology[Text Word] OR clinical study[publication type] OR case reports[publication type] OR clinical trial[publication type] OR clinical trial, phase i[publication type] OR clinical trial, phase ii[publication type] OR clinical trial, phase iii[publication type] OR clinical trial, phase iv[publication type] OR comparative study[publication type] OR controlled clinical trial[publication type] OR evaluation study[publication type] OR meta-analysis[publication type] OR multicenter study[publication type] OR observational study[publication type] OR pragmatic clinical trial[publication type] OR randomised controlled trial[publication type] OR review[publication type] OR systematic review[publication type] OR twin study[publication type] OR validation study[publication type] OR non randomised trial[text word] OR non randomised trial[text word] OR randomization experiment OR randomisation experiment OR natural experiment OR synthetic control)*

### Text mining

#### Dictionary

A manually engineered dictionary that comprised of terms on study designs was used. The scope of the dictionary involved experimental (e.g., trials), observational (e.g., cross-sectional) and secondary (e.g., meta-analysis) study designs. A total of 134 terms were included (Table 1, Supplementary material).

#### Rule based text mining approach

We designed and implemented a python algorithm to randomly select a sample of 100 abstracts to serve as a training set. The set was annotated by two authors with epidemiological and public health background (GK, TB) for existing study designs. We calculated the inter-annotator agreement as the absolute agreement rate with a value of 100.0% suggesting reliable annotations [30].

Rules were based on common syntactical patterns observed in the text that suggest the presence of a study design. The syntactical patterns make use of: (a) frozen lexical expressions as anchors for certain elements built through specific verbs, noun phrases, and prepositions, and (b) semantic place holders which can be identified through the dictionary application that suggests a study design.

In the following example of a syntactical pattern (“*we conducted a****cross-sectional study***”), to identify the study design (“cross-sectional”), the semi frozen lexical expression “we conducted a” is matched via a regular expression containing variations of such terms (e.g., conducted, performed); and “cross-sectional” gets a match through the study design dictionary. More than one syntactical patterns may be matched in an abstract referring to one or more study design mentions (which can be duplicates).

An additional (i.e., development) set with 100 randomly selected abstracts was also used to optimise the performance of the rules. A total of 20 rules were crafted (Table 2, Supplementary material shows some rule examples). General Architecture for Text Engineering (GATE) [31] was selected to implement the rules and annotate the study design mentions in the training and development sets. The observed syntactical patterns were converted into rules via the Java Annotations Pattern Engine (JAPE), a pattern matching language for GATE.

#### Data standardization and abstract level unification

To enable statistical analysis, the extracted study designs were standardised based on the Ontology of Clinical Research [32]. In cases where more than one (different) mention of study design was extracted in one abstract, we chose the lengthiest; we assumed that the longer the study design is, the more informative (i.e., most comprehensive) it is (e.g., “randomised double blinded controlled trial” against “randomised controlled trial”). After manually inspecting the training and development sets, no information loss was noted.

Domain experts (GK, IB, TB) created a classification schema for the selected study designs that involved four high-level nodes: observational, review, trial and meta-analysis. Any study designs that bore ambiguous meaning or did not have enough detail to warrant a classification (e.g., “analytical study”, “systematic approach”) were assigned into an additional category as miscellaneous. Each one of the four high level nodes has a number of lower level study designs. To prevent any information loss from the standardization process, we created also a list of common attributes – words (e.g., “community based”, “clinical”, “single blinded”, “retrospective”) used to describe the lower level study designs in the abstract text (Table 1).

**Table 1:** Classification schema of epidemiological study designs and their respective attributes

Study design	Lower level study designs	Attributes
Observational	Descriptive, cohort, case-control, cross-sectional, ecological, quasi-experimental, case report, mendelian randomisation, survey	Quantitative, qualitative, prospective, retrospective, longitudinal, nested, propensity matched, before after, time series, interrupted time series, serial, secondary, survival, case series, exploratory, follow up, comparison, population based, synthetic-control, difference in differences, natural experiment
Trial	Individually randomised, cluster randomised, non-randomised, controlled, non-controlled,	Non-blinded, single blind, double blind, triple blind, phase 1, phase 2, phase 3, phase 4, two arm, multi arm, regulatory, efficacy, safety, cost-effectiveness, non-inferiority, superiority, equivalence, pilot, feasibility
Review	Systematic, scoping, meta-analytic, investigation	-
Meta-analysis	Quantitative, qualitative	Comparison
Miscellaneous	-	-

## Results

### Text mining evaluation

To measure the system’s performance at the abstract level, we considered whether study designs were correctly identified from the text. We used the standard definitions of precision, recall and F1-Score [33]. We defined True Positive (TP) as the detection of either all the correct mentions of study design or the recognition of several mentions for one study design even if the system failed to pick up some mentions in an abstract. For example, if a study design in one abstract is “prospective cohort” and there are two mentions in the text (prospective cohort, cohort study), then the detection of either one or both these mentions would be considered a TP at the abstract level with “prospective cohort” being the representative study design. A False Positive (FP) at the abstract level is the extraction of an unrelated study design mention that has not been annotated manually. A False Negative (FN) is a study design mention that was ignored by the system (and no related mentions were extracted either). For example, if an abstract contains one or more mentions of “prospective cohort” and our method ignored all of them, then at the abstract level this would be classified as a FN.

We randomly selected a sample of 100 PubMed abstracts to act as our evaluation set. At the abstract level, the returned precision and recall were 93.5% and 91.1% respectively while the F1-Score was 92.2%. (Table 2). A relatively small drop of 3.9% in F1-Score was observed from the training to the evaluation.

**Table 2:** Precision, recall and F1-Score results for the training, development and evaluation set including the number at the document level of true positives (TP), false positives (FP) and false negatives (FN)

	TP	FP	FN	Precision (%)	Recall (%)	F1-Score (%)
**Training set**	75	4	2	94.9	97.4	96.1
**Development**	62	6	3	91.1	95.3	93.1
**Evaluation set**	72	5	7	93.5	91.1	92.2

### Query results

**Fig. 1:**
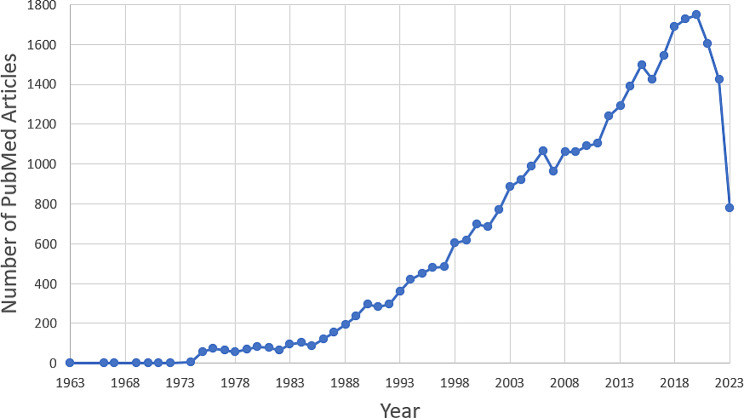
Number of published articles (*n* = 34,481) in PubMed related to epidemiological criminology from 1963 to 2023

A total of 34,481 epidemiological criminology study abstracts were returned from the query with the earliest study recorded in 1963 (Fig. 1). 13,671 (39.6%) study abstracts had an extracted study design, with the most common being observational at 37.3% (5101) followed by review (4187; 30.6%). Experimental research (i.e., trial) was present in 16.9% (2319) of study abstracts with meta-analysis at 1.4% (190). Miscellaneous study designs were noted in 13.7% (1874) of abstracts.

The most common type of lower level study design was systematic review (10.5%; 1432) followed by randomised controlled trial (8.3%; 1136), case report (5.9%; 806), cross-sectional (4.6%; 634), and cohort (4.6%; 626) (Table 3).

From 2,319 trial study designs, 18.9% (439) had the attribute double blind (49.9%; 149) followed by pilot (20.3%; 89), and phase II (12.5%; 55). The least reported attribute was phase IV (0.7%; 3) and triple blind (0.2%; 1). However, 44.5% (2274) of observational research studies had at least one recorded attribute with retrospective (42.8%; 974) and comparison (31.3%; 712) being the most commonly reported (Table 4).

**Table 3:** Top 20 most frequent lower level study designs in an epidemiological criminology PubMed abstract data sample (*n* = 13,671) from 1963 to 2023. Note: A study design can have more than one attribute

Lower level study design	High level study design	Number of PubMed abstracts	%
Systematic	Review	1432	10.5
Randomised controlled	Trial	1136	8.3
Case report	Observational	806	5.9
Cross sectional	Observational	634	4.6
Cohort	Observational	626	4.6
Randomised	Trial	611	4.5
Scoping	Review	202	1.5
Descriptive	Observational	157	1.1
Case control	Observational	123	0.9
Controlled	Trial	79	0.6
Survey	Observational	71	0.5
Cluster randomised controlled	Trial	60	0.4
Quasi experimental	Observational	52	0.4
Cluster randomised	Trial	47	0.3
Meta-analytic	Review	31	0.2
Nonrandomised controlled	Trial	23	0.2
Systematic scoping	Review	21	0.2
Cross sectional descriptive	Observational	18	0.1
Ecological	Observational	13	0.1
Nonrandomised	Trial	12	0.1

**Table 4:** Top ten most commonly used attributes to describe trial designs (*n* = 439) and observational research (*n* = 2274) in a sample of PubMed epidemiological criminology abstracts from 1963 to 2023

Trial attributes	Frequency	%	Observational attributes	Frequency	%
Double blind	149	49.9	Retrospective	974	42.8
Pilot	89	20.3	Comparison	712	31.3
Phase II	55	12.5	Prospective	633	27.8
Single Blind	49	11.2	Qualitative	170	7.5
Phase III	49	11.2	Case series	154	6.8
Phase I	30	6.8	Population based	76	3.3
Cost effectiveness	25	5.7	Follow up	75	3.3
Feasibility	17	3.9	Exploratory	64	2.8
Two arm	16	3.6	Quantitative	48	2.1
Superiority	11	2.5	Nested	14	0.6
Non inferiority	11	2.5	Survival	13	0.6
Efficacy	8	1.8	Time series	7	0.3
Equivalence	3	0.7	Interrupted time series	7	0.3
Phase 4	3	0.7	Natural experiment	6	0.3
Triple blind	1	0.2	Serial	4	0.2
			Propensity matches	1	< 0.1

### Aligning extracted study designs against the hierarchy of scientific evidence

**Fig. 2:**
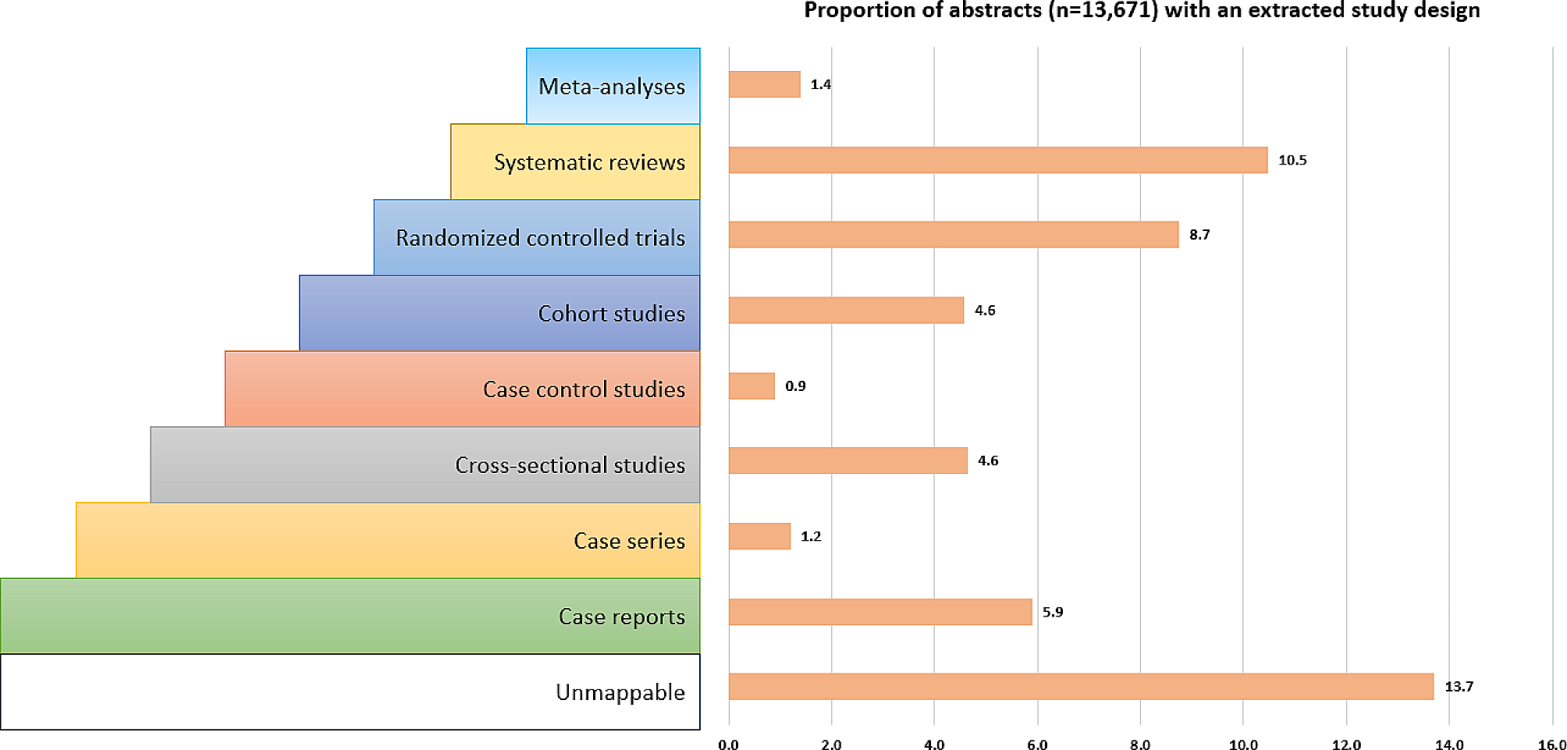
Proportion of extracted and standardised study designs aligned with the current hierarchy of scientific evidence

We used the most up-to-date hierarchy of scientific evidence [26, 27] to map the extracted and standardised study designs. Those study designs which could not be directly mapped to the hierarchy, were classified as “unmappable” (Fig. 2). Most of the studies were of observational research (17.2%; 2343) followed by studies (13.7%; 1874) with an ambiguous study design (e.g., randomised design, clinical study) and systematic reviews (10.5%; 1432). Randomised controlled trials (including cluster randomised controlled trials) represented 8.7% (1196) of reported study designs while meta-analysis accounted for only 1.4% (190) of study designs.

## Discussion

This study demonstrated that it is possible to identify study designs from a large corpus of epidemiological criminology abstracts employed by researchers using a simple rule-based text mining method. This potentially allows a reflection on both the quality of the designs employed by researchers in a whole discipline and the identification of gaps arising from this in terms of methodologies used.

Overall, observational research was most common representing 37.3% (5101) of studies, followed by reviews (4187; 30.6%), and trials at 16.9% (2319). Randomised control trials represented 8.7% (*n* = 1136) of study designs. The results suggest that many research questions in this area rely on observational research [7] rather than more rigorous designs such as clinical trials. In addition, the ability to conduct systematic reviews as well as meta-analyses requires a large and sufficient body of published literature on related research priorities and implemented interventions need to be available.

However, only 39.6% (13,671) of abstracts had an identifiable study design. Previous studies have shown that PubMed epidemiological abstracts often lack information on key characteristics such as study designs and research themes [16, 17, 34]. This lack of adequate and standardised description of the research approach along with challenges related to the conduct of quality research (e.g., hard to access population, security barriers, enhanced ethics approval processes, isolated locations) hampers the ability to perform systematic reviews and most importantly, meta-analysis on published research which can potentially lead to improving research translation, fill in knowledge gaps, improve health outcomes for offenders, and promote future research [35, 36].

Since we included a broad range of study designs, ranging from the relatively strict reporting structure of a clinical trial to the informal style of observational research, it is not surprising to note that some articles (13.7%; 1874) did not explicitly state their implemented methodology in the abstract text with studies on PubMed samples reporting similar conclusions [16, 17]. Although the abstracts featured elements of study designs in the text, even when inspected by an expert to determine their design, they are prone to subjective interpretation. For example, if there is a control group, this could be a clinical trial or a case control study. For that reason, our methodology did not seek to extract specific traits of each study design and relied on the identification of the study design itself to avoid ambiguity.

From 13,671 abstracts, almost half (47.5%; 6506) reported attributes that further described the implemented study design. Yet among those, key attributes (e.g., single blind, equivalence) from our classification schema were shown to appear only in 1 out of 5 trial study designs (18.9%) and almost half of the observational ones (44.5%). This suggests the need for standardised reporting of study design in the discipline of epidemiological criminology under reporting guidelines such as STROBE [7], CONSORT [8], SPIRIT [9], and PRISMA [10]. As randomised controlled trials are generally regulated, their design details are more likely to be clear from the abstract text. However, the reporting of such information is also influenced by journal’s requirements. Although structured abstracts were introduced in medical research in the mid 1980s [37] offering improved and higher quality information [38], some journals still enable abstracts in free text of varying length. This could likely result in a set of abstracts not explicitly stating the study design.

**Fig. 3:**
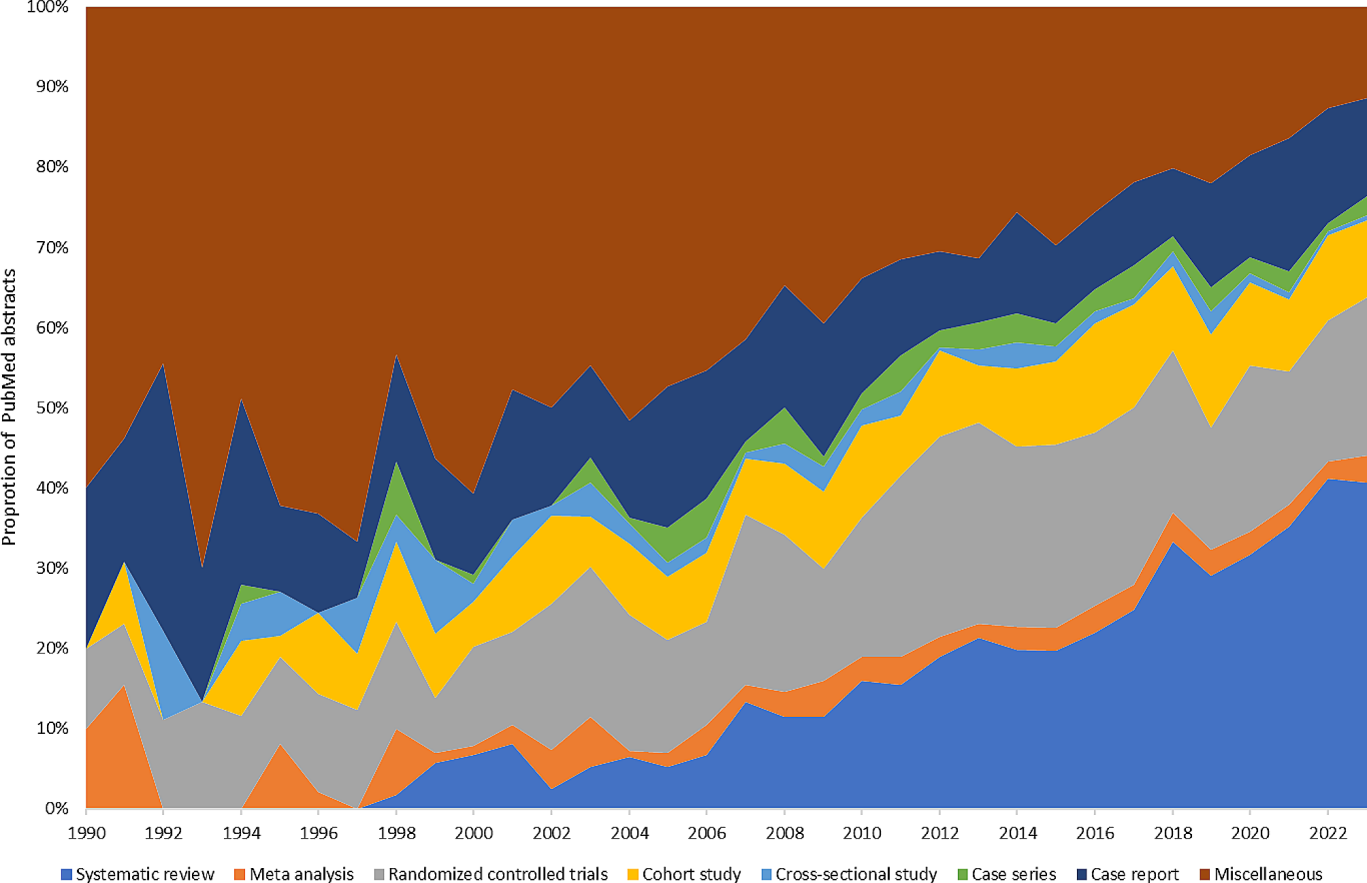
Proportion of PubMed abstracts (*n* = 13,671) with a mapped to the hierarchy of scientific evidence study design from 1990 to 2023

When mapping the standardised results against the hierarchy of scientific evidence, we found that more than one in ten abstracts (13.7%; 1874) had an ambiguous design preventing such a mapping. Mentions of “clinical”, and “analytical” studies were quite common but could not be assigned to the hierarchy of evidence. Although in the early 1990sy most studies were being of “miscellaneous” nature with 29.6%, the proportion in our sample diminished 6.3% in 2022 (Fig. 3) highlighting the improvement of reporting standards in abstract text.

Three of most important pillars of evidence in research (i.e., meta-analyses, systematic reviews and randomised controlled trials) were found to be uncommon in this field when analysing abstracts with meta-analyses representing only 1.4% (*n* = 190) of study designs. This suggests an overall poor evidence base in epidemiological criminology preventing high level evidence syntheses. The number of systematic reviews has increased since the 1990s. As our results suggest, their frequency has been exponentially increasing, especially in the last five years as others have noted [5]. Indeed in 2022 they represented 20.4% of all extracted study designs suggesting a trend towards reviews rather than more rigorous and hands on forms of research (Fig. 3). Considering the complexity of conducting research within the justice system, this is understandable. The prison setting and the isolation of its population does not foster the implementation of resource-intensive designs such as randomised controlled trials [5, 39].

This may also explain why most research (excluding the unspecified study designs) is observational in nature. The combination of case control, cross sectional, case series and case report designs amounted to 17.2% (2343) of studies, most likely due to the low cost and being easy to implement compared with randomised control trials. This aligns with epidemiological research reviews suggesting that most observational research in English speaking journals are either cohort or case control studies [40]. Although observational studies have been criticized for lacking strong clinically valid conclusions, they can detect rare or late adverse effects of treatments and indicate real-world clinical outcomes that are outside the mix of participants selected or the observations made in clinical trials [41].

Our results indicate the need for higher quality evidence with this marginalized population to improve health outcomes. Basing research priorities on results derived from methods that are known to have a relatively weak level of evidence hampers generalizability and translation into policy [7]. While randomised controlled trials were not common (8.7% of all extracted designs), an increase was observed after 2010 with more than 10.0% of abstracts reporting such a design. However, since we examined unique PMIDs, it is possible that the frequency numbers for trials presented here might be inflated as complex trials tend to produce multiple publications from the same study. Nevertheless, meta-analyses which draw on well conducted trials accounted for 1.0–2.0% of the total studies per year (Fig. 3) highlighting that in epidemiological criminology, research outputs and policies have relied heavily on observational study designs.

### Text mining error analysis

The application of this method returned encouraging results (F1-Score 92.2%), with five false positives (Precision 93.5%) and seven false negatives (Recall 91.1%). Sources of false positive errors include the extraction of a previously implemented study design (e.g., “six year follow up of a randomised controlled trial [false positive]”) and analysis (e.g., “Following a qualitative analysis [false positive]”). The reason behind the increased number (as opposed to the training and development sets) of false negatives in our evaluation set was the lack of terms in our study design dictionary because we did not consider these plausible enough to describe a study design (e.g., “comprehensive”, “open”, “steady-state”) and they were not encountered before. It is possible though that in a larger evaluation dataset, more false positives (or negatives) might appear, thus the performance of our method should be interpreted with caution.

### Limitations

Our study comes with several limitations. Using PubMed abstracts might not be enough to capture an accurate picture for offending and incarcerated populations as government articles and internal reports in this area are often not published in academic journals and studies with a more sociological and criminal focus are unlikely to appear in PubMed journals. Thus, it is possible that our current data sample underestimates the total number of research outputs in this area.

Our focus on English written abstracts could have provided potentially a different picture on the implemented study designs within this area and the inclusion of non-English articles could help ensure greater generalizability and reduce bias [42]. Although trials were the third most reported high-level design (16.9%; 2319), these numbers might be over-represented in our findings since large complex trials often have multiple publications.

We demonstrated that not all abstracts report their implemented study designs. Despite a reliable performance from our method, the number of identified study designs could be under-represented. Including full-text studies might provide a more complete picture towards the reporting of key information such as study designs within the area of epidemiological criminology. It would be interesting to explore whether if applying this method into full-text articles would improve the extraction performance and return different results.

## Conclusions

Our study demonstrated that it is feasible to extract reported study designs from a large-scale sample of PubMed abstracts to provide a high-level examination of study methods in a discipline using a simple rule-based text mining approach. However, our findings highlight that among those abstracts that reported their study design, most research on incarcerated and offending populations rely on observational methods with few clinical trials which is reflected in low numbers of meta-analyses. The yearly consistency of study types demonstrates that additional modes of research are required to address the health needs of this subgroup. Based on our findings, we encourage journals to require an accurate description of the study design in the abstract to allow the reader to quickly determine the type of study design employed. This should also be picked up in the peer review process.

### Electronic supplementary material

Below is the link to the electronic supplementary material.

Supplementary Material 1

Supplementary Material 2

## Data Availability

The datasets used in this study can be downloaded from PubMed by implementing the authors’ query.

## Abbreviations

CONSORT: Consolidated Standards of Reporting Trials
FN: False Negative
FP: False Positive
GATE: General Architecture for Text Engineering
JAPE: Java Annotations Pattern Engine
PRISMA: Preferred Reporting Items for Systematic Reviews and Meta-Analyses
SPIRIT: Standard Protocol Items: Recommendations for Interventional Trials
STROBE: STrengthening the Reporting of OBservational studies in Epidemiology
TP: True Positive
