# Supplementary material for: An analysis of published study designs in PubMed prisoner health abstracts from 1963 to 2023: a text mining study
Source: BMC Med Res Methodol. 2024 Mar 17;24:68. doi: 10.1186/s12874-024-02186-6 (PMC10944606; doi:10.1186/s12874-024-02186-6)
Supplement: Supplementary file 1 — Supplementary Material 1 [file 12874_2024_2186_MOESM1_ESM.docx]

**Additional files**

**Table 1.** List of terms used to describe study designs in epi-criminology.

| before after | feasibility | noncontrolled | regulatory |
| --- | --- | --- | --- |
| case control | follow up | non-controlled | retrospective |
| case reference | follow-up | non-inferiority | safety |
| case report | historic | nonrandomised | scoping |
| case series | historical | non-randomised | secondary |
| case-control | individually randomised | nonrandomised | serial |
| case-reference | individually randomised | non-randomised | single blind |
| clinical | interrupted time series | observational | single blinded |
| cluster randomised | investigation | phase 1 | single-blind |
| cluster randomised | literature | phase 2 | single-blinded |
| cohort | longitudinal | phase 3 | superiority |
| community based | longitudinal panel | phase 4 | survival |
| community-based | mendelian randomisation | phase i | synthetic control |
| comparative | mendelian randomization | phase ii | synthetic controlled |
| comparison | meta analyses | phase iii | synthetic-control |
| control | meta analysis | phase iv | synthetic-controlled |
| controlled | meta analytic | pilot | systematic |
| correlational | meta-analyses | population based | time series |
| cost effectiveness | meta-analysis | population-based | triple blind |
| cost-effectiveness | meta-analytic | pragmatic | triple blinded |
| cross sectional | multi arm | propensity matched | triple-blind |
| crossectional | multi center | prospective | triple-blinded |
| cross-sectional | multi study | qualitative | twin |
| descriptive | multiarm | quantitative | two arm |
| difference in differences | multi-arm | quasi experimental | two-arm |
| double blind | multicenter | quasi experimental field | validation |
| double blinded | multi-center | quasi-experimental | |
| double-blind | multi-study | quasi-experimental field | |
| double-blinded | natural experiment | questionnaire | |
| ecological | nested | questionnaire based | |
| efficacy | nested case | questionnaire-based | |
| equivalence | non controlled | randomisation experiment | |
