# Supplementary material for: An analysis of published study designs in PubMed prisoner health abstracts from 1963 to 2023: a text mining study
Source: BMC Med Res Methodol. 2024 Mar 17;24:68. doi: 10.1186/s12874-024-02186-6 (PMC10944606; doi:10.1186/s12874-024-02186-6)
Supplement: Supplementary file 2 — Supplementary Material 2 [file 12874_2024_2186_MOESM2_ESM.docx]

**Table 2.** Rule examples for the identification of study designs from PubMed abstracts. {Token.string==~"(?i)conducted|performed|did|orchestrated|organized|organised"} matches any of the words “conducted”, “performed”, “did”, “organized”, “orchestrated” or “organised” in lower or upper case. Similarly {Token.string==~"(?i)a|an"} matches either “a” or “an”, {Token.string==~"(?i)review|study|design|trial|analysis|survey|comparison|approach|research"} matches all the words within the brackets, {Token.string==~"(?i)review|trial"} matches “review” or “trial” and {Token.string==~"(?i)this"} matches “this”. (study_adjectives)? Matches when present one or more of the 134 study designs.

| **Rule** | {Token.string==~"(?i)conducted\|performed\|did\|orchestrated\|organized\|organised"} | {Token.string==~"(?i)a\|an"} | (study_adjectives) | {Token.string==~"(?i)review\|study\|design\|trial\|analysis\|survey\|comparison\|approach\|research"} |
| --- | --- | --- | --- | --- |
| **Example** | conducted | a | Randomised controlled clinical | trial |
|  | | | | |
| **Rule** | {Token.string==~"(?i)this"} | (study_adjectives)? | {Token.string==~"(?i)review\|trial"} | |
| **Example** | This | systematic | review | |
